# Can complex segmentectomies be simplified?

**DOI:** 10.1093/icvts/ivac074

**Published:** 2022-05-02

**Authors:** Dominique Gossot, Agathe Seguin-Givelet

**Affiliations:** 1 Thoracic Department, Curie-Montsouris Thorax Institute—Institut Mutualiste Montsouris, Paris, France; 2 Paris 13 University, Sorbonne Paris Cité, Faculty of Medicine SMBH, Bobigny, France

**Keywords:** Lung cancer, Sublobar resection, Segmentectomy, Intersegmental vein, Video assisted thoracic surgery

As pointed out by the article of He *et al.* [1], interest in sublobar resections (SLR) is growing. It will be boosted by the JCOG 0802 trial, the results of which are in press at the time of writing this editorial. What we already know is that, for patients operated on for a cT1a-b stage tumour, the study demonstrates a significant superiority on survival in the segmentectomy group. However, if surgical teams progressively move towards the treatment of early-stage non small cell lung cancer (NSCLC) by SLR, they will theoretically have to master any type of anatomical segmentectomy. It is accepted that segmentectomies can be divided into ‘simple’ and ‘complex’ depending on whether only one or more intersegmental planes need to be divided [2], but these so-called complex segmentectomies are even more so when they involve lower lobes. As pointed out by many authors, these procedures are a challenge [3–6]. This challenge is both technical and technological. It is partly explained by (i) the numerous anatomical variations of the segmental vessels and bronchi, which require preoperative 3D modelling [7] and (ii) the pyramidal shape of the lower lobes, which makes splitting of the intersegmental planes delicate [8].

Therefore, the article by He *et al.* [1] published in this issue is opportune because it proposes a technique based on the primary identification of the intersegmental vein (Inter-SVs). This work is based on a series of 46 patients operated on with the intention to treat, making it one of the largest experiences of posterobasal segmentectomies, i.e. S^10^ or S^9 + 10^ or S^10^ extended to an adjacent subsegment.

The posterior approach of the posterior basal segments had already been proposed by Endoh *et al.* [9] with the aim of avoiding opening the fissure. The authors described a technique of posterior dissection between the V^6^ vein and the basilar veins, allowing the identification of the B^10^ or B^9 + 10^ bronchi, then the A^10^ or A^9 + 10^ arteries. No doubt this technique is attractive because it avoids dissection in the fissure, which can be difficult and a cause of air leaks. It also avoids the creation of a tunnel between segments 6 and 10 [10]. The aim of this trick is to separate segment 6 from segment 10 to expose the arterial anatomy. More recently, Pu *et al.* [5] reported a similar technique, so-called stem branch. The procedure is initiated through an inferior pulmonary ligament approach. The basal segmental vein and bronchus are dissected, followed by their branches. Then, the target arteries are identified, based on their positional relations on CT or 3D modelization.

However, this approach has its drawbacks. It offers a limited view of the segmental pedicle, especially on the bronchus and the artery, which are seen in a narrow cone and in an uncommon and non-anatomical situation. The risk of anatomical confusion is possible, even with the support of a three-dimensional model. Moreover, an approach via the pulmonary ligament and the inferior pulmonary vein encourages severing of the venous branches near the inferior basilar vein. However, the latter represents the venous drainage of the posterobasal segments in only 18% of cases. The risk is therefore to compromise the venous drainage of the other basilar segments. Finally, and above all, this approach does not include the lymph nodes located in the fissure with the risk of leaving metastatic lymph nodes in place, a disadvantage mentioned by Endoh *et al.* [9]. Moreover, the authors show that in the segment group, the number of nodes removed is lower than in the lobe group [1].

Conversely, a fissure-first approach offers an anatomical view and allows a more extensive lymph node dissection, but it has its own shortcomings: a sometimes-tedious dissection and a higher risk of air leakage.

The choice of a mixed approach with the creation of a tunnel facilitates the exposure of the vascular structures, but the practical realization is not always simple with a risk of venous tear.

By giving the pros and cons of each approach, we want to stress that, contrary to lobectomies, a standard technique for complex SLR is far from having been found. Another question, and not the least, remains unanswered after reading the article by He *et al.*: should the section of the intersegmental plane be done manually along the vein, which is more anatomical and preserves parenchyma but exposes to more air leakages, or by stapling, which is safer in terms of leaks but less anatomical? The wish to simplify a complex technique is laudable, but there is still a long way to go before standardization. This is the interest of this new technical challenge that thoracic surgeons are facing.

**Conflict of interest:** Dominique Gossot and Agathe Seguin-Givelet received fees from Medtronic company for presentations in relation to this topic. Dominique Gossot is a consultant for an instrument manufacturer (Delacroix Chevalier).
